# London Trauma Conference 2014

**DOI:** 10.1186/1757-7241-23-S2-A1

**Published:** 2015-09-11

**Authors:** Leopold Salm, Pascal Avery, Flora Bird

**Affiliations:** 1 Homerton University Hospital, London, UK; 2 Chelsea and Westminster Hospital, London, UK; 3 Queens Hospital, Essex, UK

## Introduction

The London Trauma Conference (#LTC2014) & London Cardiac Arrest Symposium (#LCAS2014) have become an extraordinary international showcase for the innovation, research, talent and passion that is dedicated to the medical care of major trauma and critically ill patients. The 8^th^ London Trauma Conference did not disappoint and, in a reversal of the Royal Geographical Society's history of global exploration, it has now become the focus of an expanding annual pilgrimage of the trauma faithful from across the world. This year, 1,113 delegates representing a wide range of countries enjoyed a programme designed to challenge, inform and, true to the ethos of the hallowed corridors of the venue, pioneer new medical frontiers.

The opening two days of the Main Conference centered upon key trauma issues, day three focused upon Air Ambulance and Pre-Hospital Care, with the London Cardiac Arrest Symposium returning as a two-day event on the third and fourth days. Concurrently with the main conference, Master classes in Thoracotomy / REBOA and Cardiac Arrest, as well as Breakaway Sessions covering topics as diverse as Trauma Research, Remote Critical Care and Motorsport Medicine, provided lots of choice and difficult decisions!

Whilst there were many memorable and fascinating elements to the 2014 London Trauma Conference, if there was a theme building at this year's conference it was that we may be witnessing the beginning of an endovascular revolution in resuscitation. Extraordinary talks on REBOA in London, Pre-Hospital ECMO in Paris and the prospect of Selective Aortic Arch Perfusion (SAAP) could herald new approaches to treating the previously unsalvageable patient.

The article that follows describes highlights from the Main Conference programme and introduces the outstanding range of research abstracts that were presented at the London Trauma Conference 2014. However it would be impossible to comprehensively cover the depth and insight of the talks in this supplement but if you want to hear what you missed in 2014 and whet your appetite for December 8^th^-11^th^ 2015 ((#LTC2015) then session highlights, interviews with speakers, talk overviews and great conference summaries are available as links from the article below, with London Trauma Conference Podcasts published on sites including St. Emlyn's, Life in the fast lane and RCEM FOAMed.

## Major trauma

The two day Major Trauma programme (http://www.londontraumaconference.com/downloads2014/LTC14_programme.pdf) reads like a Who's Who of international trauma care: Professor Karim Brohi, Dr Gareth Davies, Professor Chris Moran, Professor James Manning, Professor Mauro Oddo, Professor Ben Bobrow … the list goes on. Podcast summaries of the days are available at (http://stemlynsblog.org/ltc-day-1/) (http://stemlynsblog.org/ltc-day-2/), interviews with some individual speakers are linked from the text and more detailed talk summaries can be found by clicking on the speaker names.

## Clinical concepts

**Professor Karim Brohi** opened the conference with a characteristically fascinating and dynamic talk on the recognition and management of traumatic arterial dissection, best summarized in his own words in his podcast (http://www.stemlynspodcast.org/e/karim-brohi-at-ltc-with-stemlyns/). **Mr Doug West**'s podcast follows a fascinating talk discussing the improved outcomes associated with surgical fixation of flail chest (http://www.rcemfoamed.co.uk/portfolio/ltc-podcast-1-chest-trauma/) and his less heart-warming experience of the consequences of injudicious chest drain insertion! **Dr Bob Winter** made a racing visit from his responsibilities at the concurrently running Intensive Care Society Meeting to regale the audience with stories of glamour models at his Excel Centre conference before providing a reassuringly pragmatic view on the equally heady subject of inflammation in trauma. The Peter Baskett Memorial lecture this year was given by **Dr Stephen Leadbetter**, Director of the Wales Institute of Forensic Medicine, who gave a charismatic and fascinating insight into what could be learnt from trauma deaths.

**Dr Jerry Nolan** addressed the controversial issue of cervical spine control during intubation and provided extensive evidence that all airway interventions result in some cervical spine movement, with video-laryngoscopy causing less movement but also less success when studied in the pre-hospital environment. The perennial problems of assessing haemorrhagic shock and its complex cascade of physiological sequelae were eloquently discussed by **Professors James Manning** and **Tim Harris** (http://www.stemlynspodcast.org/e/prof-tim-harris-joins-stemlyns-on-shock-assessment/), both of who spoke with authority on several subjects during the conference.

This year there was a strong focus on CNS trauma with a series of talks delivering novel concepts and expert reviews of our current state of knowledge. **Dr Mauro Oddo** provided a comprehensive update on multimodality brain monitoring following traumatic brain injury. Challenging dogma as ever, **Dr Gareth Davies** (http://www.stemlynspodcast.org/e/impract-brain-apnoea-with-gareth-davies-from-london-hems/) shone a spotlight onto the long-recognized mammalian phenomenon of impact apnoea and proposed that respiratory arrest following head injury may account for an unrecognized burden of extremely early hypoxic brain injury – perhaps established before even the most rapid of pre-hospital providers can attend. This concept not only alters conventional thinking on the aetiology of traumatic neurological injury but dovetails beautifully into **Mr Mark Wilson's** (http://www.stemlynspodcast.org/e/mark-wilson-joins-stemlyns-to-discuss-the-goodsam-app-iain-beardsell-interviews-from-the-ltc/) important and revolutionary global GoodSAM app (https://www.goodsamapp.org). This App alerts and deploys registered medical responders who are in the locality of an incident within seconds of an event and all readers of this article should consider registering. **Mark Wilson**'s entertaining review of the advances and deficits in the understanding of traumatic brain injury was not only scientifically revealing but was delivered to what must be the most creatively accomplished slide set ever given at a medical conference! This technological *tour de force* rounded off an excellent session where **Dr Andrew Jackson** discussed the current and future therapeutic options following spinal cord injury and **Mr Nick Haden** provided some clarity for the non-surgical audience members on the troublesome area of cervical spine fracture stability.

## Special populations

Talks on trauma in the elderly, paediatrics, pregnant and those foolhardy enough to participate in motorsports, provided some fascinating examples of how conventional systems and trauma management may be inadequate in these patient groups. **Dr Marius Rehn** warned of the ‘grey tsunami& of our global ageing population and how altered physiology, anatomy, comorbidity and medications can dramatically influence the response to trauma and its medical management. **Mr. Ross Fisher** made a familiar but coherently argued plea: *‘Kids are not small adults’* ! (http://www.stemlynspodcast.org/e/ross-fisher-and-natalie-may-discuss-paediatric-major-trauma-at-the-london-trauma-conference/). **Professor Tim Draycott** entertainingly addressed trauma in pregnancy with a key central message: *‘What is best for the mother, is best for the baby’* with maternal lateral tilt and early definitive airway management practical take-home lessons. **Dr Tim Moll**'s astonishing experience of trauma in motorsport described the injury patterns associated with an eye watering range of ways that a motorbike can eject its fragile rider at extreme high speed. This subject was also covered in great detail in the **Motorsports Medicine** Breakaway session that similarly emphasized the technological innovation being applied to personal protection and injury prevention – however at no point was it suggested that not riding a motorbike at 200 mph may be a solution to some of these issues.

## Trauma systems

It has been a dramatic few years in the UK with the implementation of Major Trauma Networks and **Professor Chris Moran**, the UK National Director for Major Trauma, gave his unique perspective on the best elements of trauma performance. He proposed that enhanced pre-hospital approaches, excellent leadership, military discipline, standardised care, and governance, are but some of the many reasons one is 30% more likely to survive major trauma today than 2 years ago in the UK. Expanding on one element of this service delivery, **Mr Ian Bailey** drew on his experience of developing one of the UK Major Trauma Centres to give an insight into ensuring the training and competence of trauma surgeons. Introducing technology to remotely deliver expertise, **Dr Conor Deasy** passionately proposed the benefits in clinical leadership provided by telemedicine in trauma care.

## Education and training

**Dr Matthew Wiles** took on the heretical but popular position that ATLS has had its day. He exploded false principles that have been dogmatically perpetuated and suggested that, in 3091 studies, this course designed for rural doctors had conferred no benefit to western populations. Conversely and making a strong case for mass trauma training in resource poor environments of the developing world, **Dr Doug Wilkinson** described the development of the Primary Trauma Care (PTC) Foundation. To date the PTC Foundation has trained an extraordinary 60,000 medical professionals in 60 countries. The key messages of empowering, devolving and standing back that have contributed to the success of this phenomenal initiative are fascinatingly at odds with the increasingly governed and micro-analysed teaching methods being pursued in the developed world. An extreme example of the rarified environment being developed in trauma care is illustrated by the insights of **Dr. Tom Evens** who combines a medical career with the coaching of elite level athletes. He draws close and illuminating comparisons between the psychological and training tools required to consistently perform at the highest standard in medicine and sport.

## Quickfire controversies

One of the recent format innovations at the London Trauma Conference has been to challenge a panel of trauma specialists to deliver succinct ten minute answers to thorny clinical dilemmas. **Dr Ross Davenport, Mr Mark Wilson, Dr Julian Thompson, Dr Dan Ellis, Professor Tim Harris** and **Dr Conor Deasy** each took their turn on the soapbox to deliver entertaining and highly opinionated quickfire thoughts on contentious issues.

## Air ambulance and pre-hospital care

The Norwegian Air Ambulance kindly sponsored the excellent, thought provoking and hugely inspiring programme on the third day of this year's London Trauma Conference. A podcast summary of the day is available (http://stemlynsblog.org/ltc-day-3/).

## Learning from tragedy

Undoubtedly the most harrowing lectures of the conference were the 2 brave accounts of recent air ambulance crashes, with the victims known to many of the audience. The Chief Executive of the Norwegian Air Ambulance (NAA), **Mr Syver Leivestad**, and Press Officer, **Ms Siv Tonje Solfjeld**, described the crash on 14^th^ January 2014 when an NAA helicopter collided with power lines 90 feet above ground, tragically resulted in two crew fatalities and one severely injured crew member. They movingly described the impact on NAA colleagues and the challenges in managing such an incident, including those involving the press. **Dr Stephen Herns**, then gave a first-hand account of the helicopter crash that killed 10 people in Glasgow during November 2013. He described the emotional stress of attending to an incident involving friends and colleagues, and the benefits that have been gained from using the military TRIM services, detailed reflection on events and informal debriefs in the pub with colleagues.

## Innovations

A patient group with a historically terrible prognosis is the patient exsanguinating from uncompressible pelvic haemorrhage. This group is the focus of one of the real highlights of this year's conference – the rise of endovascular resuscitation techniques - in this case the world's first pre-hospital deployments of Resuscitative Endovascular Ballooning of the Aorta (REBOA). **Dr Sam Sadek** helped lead the training programme at London's Air Ambulance that has allowed the deployment of the technique and by chance has twice already been filmed on TV successfully inserting REBOA. Despite his gathering media fame, **Dr Sadek** humbly proposed REBOA as a rapid, lifesaving but temporary, bridge to damage control surgery or interventional radiology.

In addition to the keynote speeches there were a series of brief and thought-provoking talks that the session chair amusingly referred to as ‘the London Trauma Conference's answer to speed dating’. Innovations presented included **Dr Stefan Candefjord** explaining how new microwave technology could non-invasively diagnose haemorrhagic stroke and traumatic brain injury and **Dr Nils Ooverland**, giving a convincing argument for point of care ultrasound to detect pneumothoraces. He further described a recent study using new microwave technology to assess for pneumothoraces on pig models that demonstrated a sensitivity and specificity of 100%. A larger study is planned for 2015.

## Clinical insights

Giving a characteristically entertaining and astute view from a highly governed Australian system, **Dr Stefan Mazur** irreverently discussed the transport of psychiatric, obese, and infectious patients (http://www.rcemfoamed.co.uk/portfolio/ltc-podcast-2-prehospital-uss-transfer-of-difficult-patient-prehospital-txa/). Experienced pre-hospital practitioners often reinforce the value of experience in ‘reading a scene’ but the talk by the forensic pathologist **Professor Guy Rutty** took this to a completely new level. Scene analysis to reconstruct events, determine causality, predict injury patterns and focus further investigation, together with startling imaging and forensic techniques at his disposal gave extraordinary insight into just how much it is possible to determine post mortem. Pre-hospital practitioners really need to be collaborating to learn whether there is anything else we can be doing to stop our patients dying.

In the fast and furious quickfire sessions, **Dr Per Kristian Hyldmo** challenged the value of routine C spine collar use, **Dr Jostein Hagemo** questioned whether we should be giving units of packed red blood cells without the support of other blood products, **Dr Dan Elis** tried to find the correct time to use arterial blood gases, **Dr Stefan Mazur** declared himself a tranexamic acid heretic until further studies have reported and **Associate Professor Cliff Reid** convincingly advocated apneic oxygenation during intubation; a goldmine of high quality opinions and evidence.

## Pre hospitals systems

**Professor Wolfgang Voelckel** spoke passionately about high quality trauma care but expressed concern at the national and international variability in care and advocated collaborative research, meta-analyses and international consensus to drive up universal standards. It was exactly this approach of evidence based care that **Dr Andreas Kruger** presented to support the expansion of physician manned pre-hospital care. His data sought to identify exactly when advanced care can make a difference and provided compelling arguments for far higher physician: population ratios than delivered in many systems. In addition to the appropriate number of enhanced care teams, in the UK there remains a debate on the best way to deliver the enhanced care team to the patient. **Captain Neil Jeffers** took up this theme by discussing the issues involved in UK night helicopter missions.

The difficulties in ensuring high quality care and the paucity of governance used to be criticisms levelled at Pre-Hospital Care in the UK. However in recent years it seems that the rigor and standard of training and governance in Pre-Hospital systems across the world now exceeds that that exists in hospitals. **Associate Professor Cliff Reid** is a convincing evangelist for extremely high quality education in pre-hospital care and he spoke compellingly of the Sydney HEMS training programme, concluding with the aphorism ‘*Under pressure you don't rise to the occasion, but you sink to your level of training’*.

However some would argue that this constant oversight has gone too far and that it is less necessary if a system retains their experienced doctors rather than recruit a constant stream of new juniors. This provided a fascinating context for a head to head debate between **Dr. Dan Ellis** and **Professor Marten Sandberg** on the subject of: ‘ *Clinical governance in pre-hospital care: tight systems are best’.* A robust and entertaining discussion followed with the audience voting overwhelmingly for tight clinical governance but the speakers agreeing that that clinical autonomy backed by extensive experience *and* system structure to guarantee a minimal standard of replicable care are vital to delivering high quality care.

## London Cardiac Arrest Symposium

If there was any concern that the science and challenge of managing cardiac arrest had disappeared into algorithms and protocols then the London Cardiac Arrest Symposium thrillingly rejected the notion. The two day programme (http://www.londoncardiacarrestsymposium.com/programme.html) was packed with innovation and novel approaches to improve survival in this group and highlights of the second day, held in the Main Conference venue, are discussed below.

## Innovative resuscitation techniques

The French are again demonstrating their prowess at revolution with an extraordinary and thought provoking talk by **Dr. Lionel Lamault** about pre-hospital ECMO in Paris. Part scientific innovation, part Paris tourist guide, the talk discussed the challenge of pre-hospital ECMO with images of it being performed in various locations around the city, including in Le Louvre Museum (http://www.rcemfoamed.co.uk/portfolio/ltc-podcast-6-prehospital-ecmo/). Refining the in hospital application of this technology, **Dr Tomasso Mauri** outlined key factors determining when to start ECMO including time, age, co-morbidities, and efficiency of CPR. Continuing the endovascular revolution, The Douglas Chamberlain Lecture was delivered this year by **Professor James Manning**, on Selective Aortic Arch Perfusion (SAAP). He outlined this exciting resuscitation technique; inflating a balloon in the descending thoracic aorta to divert blood to supply the heart and brain, maximizing coronary perfusion and minimising neurological injury.

## Cardiopulmonary Resuscitation (CPR)

A considerably more invasive alternative to these endovascular techniques was discussed by **Professor Tim Harris** who explained how much more effective open chest cardiac massage is than conventional compressions and proposed that it delivers improved coronary perfusion pressures and return of spontaneous circulation rates. At the opposite end of the invasive spectrum, **Professor Bob Bobrow** argued that, if early bystander CPR can more than double the odds ratio for survival in out-of-hospital cardiac arrest and that studies are ambiguous on the benefit of rescue breaths, minimizing the barrier to commencing CPR by non-medical professionals by training Compression Only CPR may improve outcome.

Although **Professor Niklas Nielsen** proposed that enthusiasm for therapeutic hypothermia has undoubtedly cooled in the light of recent evidence, **Dr. Peter Paal** discussed the contributory role of accidental hypothermia in survival following prolonged burial and cardiac arrest in avalanche. **Professor Charles Deakin** addressed the controversies of the optimal airway in cardiac arrest and, whilst concluding that the literature was inconclusive, did advocate that mechanical ventilators avoid the risks of hyperinflation that adrenalized resuscitators are prone to deliver manually. **Dr Peter Paal** spoke a second time to discuss the use of monitoring in cardiac arrest including the range of non-invasive techniques such as ETCO_2_, NIRS (near infra-red spectroscopy) and ultrasound.

In a subject that is increasingly dominated by technology, it was refreshing to hear **Professor Richard Schilling** advocating the importance of an old friend in VF cardiac arrest – the beta blocker. He suggested that beta blockers have a myocardial protective effect, are the only anti arrhythmic to have any prognostic benefit after MI, and as a result, their use may translate to benefit in early management of cardiac arrest.

## Post resuscitation care

**Dr NarbehMelikian** tackled the thorny question of which patients in arrest or post ROSC should go directly to PCA and **Professor Mauro Oddo** reviewed the extensive and conflicting literature on prognostication post cardiac arrest before presenting a recent trial of a multimodal prognostication tool using brainstem testing, early EEG, and neuron specific enolase which achieved 0.89 specificity.

## London Trauma Conference masterclasses and breakaways

Concurrent with the Main Conference Programme there were an excellent range of smaller sessions covering specialist topics over the 4-day conference. The delegate feedback scores for these sessions were outstanding and, in response to requests from previous years, several new masterclass programmes were added this year.

(http://www.londontraumaconference.com/breakaways.html).

Masterclass days were organized for professional groups including Trauma **researchers, junior doctors, surgeons, nurses and advanced paramedics**. (http://www.rcemfoamed.co.uk/portfolio/ltc-podcast-4-trauma-nursing-research/) and for specific clinical topics such as **Thoracotomy/REBOA** and **Cardiac Arrest**.

Enthusiasts of dangerous sports and wild places were extremely positive about two new sessions added this year: **Remote Critical Care** and **Motorsport Medicine** (http://www.rcemfoamed.co.uk/portfolio/ltc-podcast-3-25-years-between-through-and-over-the-hedges-of-ireland/).

## Research and prizes

In addition to new research presented by the invited speakers, close to 50 abstracts were submitted for presentation at the London Trauma Conference 2014. The 44 selected abstracts are published in this supplement, with 35 posters on display for the four days of the conference. 9 authors bravely took up the challenge to make an oral abstract presentation in the unconventionally informal ‘Stand up science’ session.

